# Sodium-glucose cotransporter 1 promotes the biofunctions of perivascular preadipocytes mediated by Akt/mTOR/p70S6K signaling pathway

**DOI:** 10.1152/ajpcell.00606.2023

**Published:** 2024-04-22

**Authors:** Zhiquan Liu, Jiayu Wang, Peiqing Tian, Yixuan Liu, Liyun Xing, Caihua Fu, Xianwei Huang, Ping Liu

**Affiliations:** ^1^Department of Cardiology, The Second Hospital of Shandong University, Jinan, China; ^2^Division of Life Sciences and Medicine, Department of Cardiology, the First Affiliated Hospital of USTC, University of Science and Technology of China, Hefei, China; ^3^Division of Cardiology, Beijing Anzhen Hospital, Capital Medical University, Beijing, China; ^4^Department of Cardiology, Jinan Central Hospital Affiliated Shandong University, Jinan, China; ^5^Department of Emergency, The First Affiliated Hospital of Xiamen University, Xiamen, China

**Keywords:** adipogenesis, preadipocyte, SGLT-1, sodium-glucose cotransporter 1, vascular remodeling

## Abstract

The influence of SGLT-1 on perivascular preadipocytes (PVPACs) and vascular remodeling is not well understood. This study aimed to elucidate the role and mechanism of SGLT-1-mediated PVPACs bioactivity. PVPACs were cultured in vitro and applied ex vivo to the carotid arteries of mice using a lentivirus-based thermosensitive in situ gel (TISG). The groups were treated with Lv-SGLT1 (lentiviral vector, overexpression), Lv-siSGLT1 (RNA interference, knockdown), or specific signaling pathway inhibitors. Assays were conducted to assess changes in cell proliferation, apoptosis, glucose uptake, adipogenic differentiation, and vascular remodeling in the PVPACs. Protein expression was analyzed by Western blotting, immunocytochemistry, and/or immunohistochemistry. The methyl thiazolyl tetrazolium (MTT) assay and Hoechst 33342 staining indicated that SGLT-1 overexpression significantly promoted PVPACs proliferation and inhibited apoptosis in vitro. Conversely, SGLT-1 knockdown exerted the opposite effect. Oil Red O staining revealed that SGLT-1 overexpression facilitated adipogenic differentiation, while its inhibition mitigated these effects. ^3^H-labeled glucose uptake experiments demonstrated that SGLT-1 overexpression enhanced glucose uptake by PVPACs, whereas RNA interference-mediated SGLT-1 inhibition had no significant effect on glucose uptake. Moreover, RT-qPCR, Western blotting, and immunofluorescence analyses revealed that SGLT-1 overexpression upregulated FABP4 and VEGF-A levels and activated the Akt/mTOR/p70S6K signaling pathway, whereas SGLT-1 knockdown produced the opposite effects. In vivo studies corroborated these findings and indicated that SGLT-1 overexpression facilitated carotid artery remodeling. Our study demonstrates that SGLT-1 activation of the Akt/mTOR/p70S6K signaling pathway promotes PVPACs proliferation, adipogenesis, glucose uptake, glucolipid metabolism, and vascular remodeling.

**NEW & NOTEWORTHY** SGLT-1 is expressed in PVPACs and can affect preadipocyte glucolipid metabolism and vascular remodeling. SGLT-1 promotes the biofunctions of PVPACs mediated by Akt/mTOR/p70S6K signaling pathway. Compared with caudal vein or intraperitoneal injection, the external application of lentivirus-based thermal gel around the carotid artery is an innovative attempt at vascular remodeling model, it may effectively avoid the transfection of lentiviral vector into the whole body of mice and the adverse effect on experimental results.

## Graphical abstract

**Figure:**
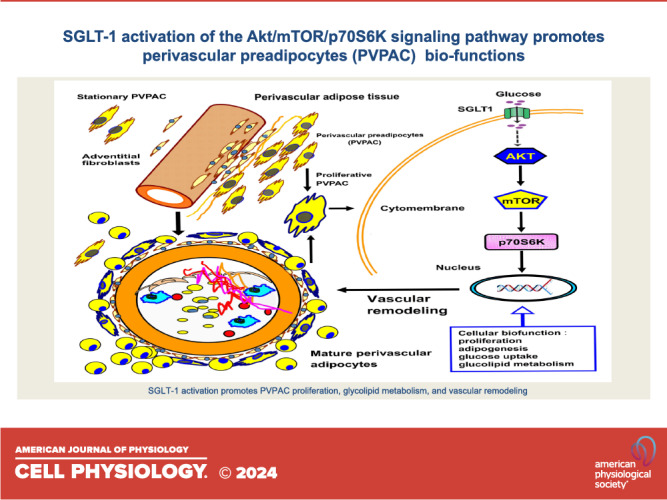

## INTRODUCTION

The sodium-glucose cotransporter (SGLT) encoded by SLC5A serves as a cotransporter for glucose and sodium, playing a pivotal role in regulating glucose metabolism in vivo (1–3). Notably, there are six subtypes of SGLT, with sodium-glucose cotransporter 1 (SGLT-1) and sodium-glucose cotransporter 2 (SGLT-2) being the most abundant and extensively investigated (4). SGLT-2 exclusively resides in the luminal membrane of the proximal tubule (5), whereas SGLT-1 is widely distributed in the small intestine, kidney, heart, adipose tissue, and other bodily regions (5, 6). Despite the remarkable advantages of SGLT-2 inhibitors in hypoglycemic and cardiorenal protection, extensive investigations have focused on SGLT-2 protein (7), and the precise role of SGLT-1 in these tissues remains to be established.

Preclinical and initial clinical trials have demonstrated that SGLT-1 inhibitors, either alone or in combination with SGLT-2 inhibitors, effectively ameliorate postprandial hyperglycemia in patients with diabetes (8), underscoring the indispensable role of SGLT-1 in glucose metabolism. Notably, recent findings revealed that SGLT-1 and SGLT-2 inhibitors, such as sotagliflozin, ameliorate left atrial remodeling and electrical remodeling in metabolic heart failure with preserved ejection fraction (HFpEF) (9). Garcia-Ropero et al. (10) proposed that SGLT-1 participates in pathological processes, such as glycolipid metabolism, oxidative stress, myocardial ischemia, and heart failure (HF). However, in contrast to the enthusiastic research on SGLT-2, minimal attention has been directed toward SGLT-1, let alone its mechanism of action in adipocyte biological function and vascular remodeling.

Perivascular adipose tissue (PVAT) enveloping the viscera of a centrally obese body is implicated in inducing inflammation, atherosclerosis, and vascular remodeling (11–13). Comprising preadipocytes, adipocytes, fibroblasts, stem cells, immune cells, and neurons, PVAT not only provides mechanical support to the vasculature but also releases various cytokines in a paracrine and endocrine manner to regulate vascular tone and function (12, 13). Angueira et al. (14) contended that PVAT originates from fibroblast progenitors and preadipocytes. Preadipocytes, the predominant cellular constituent of PVAT, represent the active form of adipocytes with a robust capacity for fat formation (11, 14). Consequently, preadipocytes and adipocytes have garnered increasing attention because of their pivotal roles in the biological function of PVAT.

Adenosine monophosphate-activated protein kinase (AMPK) plays a pivotal role in maintaining the equilibrium of cellular energy metabolism (15). Akt, also known as protein kinase B, is a proto-oncogene implicated in the regulation of diverse cellular functions, including metabolism, growth, proliferation, survival, transcription, and protein synthesis (16). Furthermore, mammalian target of rapamycin (mTOR) plays a critical role in modulating autophagy induction and serves as a major inhibitor of autophagy occurrence (17). Consequently, the AMPK-related pathway is a crucial component of the eukaryotic signal transmission network that governs cytoskeletal maintenance, cell survival, regulation of cell proliferation and differentiation, and is involved in cell emergencies and apoptosis under both normal and pathological conditions (17). However, the precise interactions between AMPKs and SGLT, as well as their regulatory influence on the biological functions of preadipocytes and adipocytes, remain unclear.

Drawing on previous investigations, we used lentiviral vectors to induce overexpression and underexpression of the SLC5A1 gene. This approach allowed us to explore the effects of SGLT-1 protein interaction with AMPK, Akt, and mTOR on the biological functions of perivascular preadipocytes (PVPACs).

## MATERIALS AND METHODS

### Materials

The cell culture reagents were procured from Biological Industries (BI, Israel). Primary antibodies used for Western blot analysis were acquired from Abcam (Cambridge, UK) and included SGLT-1 (1:1,000, #ab14686), glucose transporter 1 (GLUT-1, 1:1,000, #ab115730), glucose transporter 4 (GLUT-4, 1:1,000, #ab33780), FABP4 (1:1,000, #ab92501), and VEGF-A (1:1,000, #ab46154). Antibodies from CST (Beverly, MA) were also used, namely, p-AMPKα (Thr172) (1:1,000, #2535), p-Akt (Ser473) (1:1,000, #9271), p-mTOR (Ser2448) (1:1,000, #5536), and p-p70s6k (Thr389) (1:1,000, #9234). The β-actin antibody (20536-1-AP) was sourced from Proteintech (Wuhan, China). Secondary antibodies were obtained from ZSGB-Bio Ltd. (Beijing, China). PVDF membranes were procured from Millipore Corporation (Beijing, China). ECL detection reagents and methyl thiazolyl tetrazolium (MTT) were purchased from Beyotime Biotechnology (Shanghai, China). The PrimeScript RT reagent kit with gDNA Eraser was purchased from Takara Bio, Inc. (Shiga, Japan). MK-2206, rapamycin, Pluronic F-127 (P2443), and poloxamer 188 (P5556) were obtained from Sigma-Aldrich Corporation (St. Louis, MO). Other reagents were sourced from Invitrogen (Carlsbad, CA) and Solarbio Ltd. (Beijing, China).

### Isolation, Culture, and Identification of Preadipocytes

All animal procedures conducted in this study were approved by the Research Ethics Committee of The Second Hospital of Shandong University (Ethical No.: KYLL-2022LW057) and adhered to the ethical principles outlined in the 1975 Declaration of Helsinki, along with the animal usage guidelines established by Shandong University.

The isolation, cultivation, and characterization of preadipocytes from PVAT were performed in accordance with previously documented protocols (18). Male C57BL/6J mice, aged 2–3 wk, were euthanized by intraperitoneal injection of 1% pentobarbital sodium. The thoracic aortic ring, ∼3–4 mm in length, was rigorously dissected and submerged in a phosphate-buffered saline (PBS) solution. Subsequently, the intima, media, and adventitia of the aortic rings were mechanically removed under a microscope to obtain a pure PVAT. This PVAT was further sectioned into small fragments and digested using collagenase A (1 mg/mL) in Krebs-Ringer bicarbonate buffer (pH 7.4) supplemented with 4% (wt/vol) fatty acid-free bovine serum albumin (BSA, fraction V), and 5.5 mmol/L glucose. Digestion was performed at 37°C in a humidified atmosphere containing 5% CO_2_, with continuous vigorous shaking at 60 cycles/min. Subsequently, the fat cells were dispersed, and collagenase was eliminated by passing the mixture through a 150-μm mesh with Krebs-Ringer bicarbonate buffer. The resulting cells were washed three times and centrifuged at 135 *g* for 3 min. The uppermost floating layer, which consisted of unilocular adipocytes, was collected. To prevent stromal cell contamination, dissociated fat cells underwent repeated pipetting, followed by multiple washes with PBS, and were centrifuged at least three times.

The isolated cells were then seeded in 25 cm^2^ culture flasks (Sigma-Aldrich, St. Louis, MO), cultured in complete Dulbecco’s modified Eagle’s (DMEM) medium supplemented with 15% fetal bovine serum, and incubated at 37°C with 5% CO_2_. Cells floated and adhered to the upper inner surface of the flask. After 6–7 days, the medium was aspirated and the flasks were inverted. Medium was occurred every 2–3 days until the cells reached confluence. Following cell passage, experiments were conducted before passage 6. For cell identification and assessment of PVPACs purity, monoclonal antibodies specific for α-actin (1:200) and vimentin (1:200), along with secondary antibodies conjugated to TRITC (1:100), were used for immunocytochemistry. Subsequently, cultured PVPACs were transfected with various stimulants.

### Recombinant Lentiviral Vector Preparation and Transfection

Similar to the study conducted by Cheng et al. (19), for the analysis of overexpression, the SLC5A1 sequence was cloned into the lentiviral expression vector GV492 (Shanghai, China). Lentivector Expression Systems were used to generate lentiviruses (1 × 10^9^ TU/mL). Mouse preadipocytes were plated at a density of 5 × 10^3^ cells/well in 96-well plates. Following a 24-h incubation period, the cells were transduced with lentivirus, consisting of 10 μL of recombinant SLC5A1 lentiviral stocks or control lentiviral stocks, along with 90 μL of complete medium. After 12 h of incubation, the medium was replaced with fresh complete medium. Approximately 3–4 days later, the infection rate of the cells was directly visualized through the expression of green fluorescent protein, as observed under fluorescent microscopy. The infected cells were then detached from the wells and transferred to a 100-mm cell culture dish, where they were cultivated under drug pressure with 10 μg/mL puromycin for a duration of 2 wk until stable clones became apparent. These stable monoclonal cell lines were subsequently seeded into 24-well plates and cultured in a medium supplemented with 5 μg/mL puromycin to facilitate amplification. Selection of SLC5A1 overexpression cell lines (designated as Lv-SGLT1) was performed through RT-qPCR analysis. Control cell lines (designated as Lv-GFP) were generated following the same protocol using a control lentivirus.

To knockdown the expression of SLC5A1, target sequences specific to SLC5A1 were used: 5′- GTGGTGAACATCAACGGTATT-3′ for RNAi (#84254-1), 5′- GCCGGATTCTATATACAGAAA-3′ for RNAi (#84255-1), and 5′- CATGAAAGCTATTCCAACTAA-3′ for RNAi (#84256-1). A scrambled control sequence, 5′- TTCTCCGAACGTGTCACGT-3′ (#CON313), was also included. These sequences were cloned into the lentiviral shRNA expression vector GV493 (Genechem, Shanghai, China) to generate a lentivirus at a concentration of 1 × 10^8^ TU/mL. Initially, three different lentiviruses with distinct target sequences were tested, revealing that RNAi (#84254-1) was the most effective in knocking down SLC5A1. Consequently, lentivirus containing this specific target sequence was selected for subsequent experiments. Mouse preadipocytes were seeded at a density of 5 × 10^4^ cells/well in 12-well plates. Following a 24-h incubation period, the cells were transfected with lentivirus, consisting of 40 μL of SLC5A1 RNAi (#84254-1) lentiviral stocks or control lentiviral stocks, along with 360 μL of complete medium. Twelve hours postinfection, the medium was replaced with a fresh complete medium. The knockdown effect of SLC5A1 was assessed by RT-qPCR. The SLC5A1 knockdown cell pool was designated as the SLC5A1 underexpression cell line (designated as Lv-siSGLT1).

### Cell Transfection and Experiment In Vitro

Following confirmation of PVPACs purity, the cells underwent synchronization of their cell cycles through a 12-h serum-starvation period. Subsequently, the cells were transfected with viral liquid (Lv-GFP, Lv-SGLT1, and Lv-siSGLT1) for 12 h, followed by treatment with puromycin dihydrochloride. To induce differentiation, the cells were subjected to our previously described “Cocktail induction method” (11). In this experiment, mouse PVPACs were categorized into the following groups: NC (negative control), Lv-GFP (GFP control), Lv-SGLT1 (overexpression), Lv-siSGLT1 (knockdown), Lv-SGLT1 + MK-2206 (Akt-specific blocker), and Lv-SGLT1 + rapamycin (mTOR-specific blocker).

### Cell Viability

The proliferative capacity of PVPACs was assessed using the MTT assay. Cells were initially seeded in a 96-well plate at a density of 1.5 × 10^4^ cells/well and left to culture overnight. Cell transfection or stimulation was initiated when PAPVCs reached a confluence of 60–70%. After 48 h of stimulation, the cell culture medium was replaced with 100 μL of fresh culture medium containing 20 μL of MTT (5 mg/mL). Following a 4-h incubation at 37°C, 150 μL of dimethyl sulfoxide (1.1 g/mL) was added to the medium, after 1-h incubation at 37°C, the absorbance at 490 nm for each well was determined using an enzyme-linked immunosorbent assay (ELISA) conducted on a Bio-Tek 311 Microtiter Plate Reader (Elx 800, Bio-Tek, Winooski, VT).

### Oil Red O Staining

On *day 10* after preadipocytes differentiation, the cells underwent a series of steps. Initially, they were washed with PBS and fixed in 10% formalin for 1 h. Following fixation, the cells were stained with a filtered Oil Red O solution for 30 min. After staining, the Oil Red O solution was carefully removed and the plates were rinsed three times with water before being allowed to dry. Stained lipid droplets were visualized and photographed using an inverted microscope (Carl Zeiss, Axiovert 200 M). To extract the intracellular Oil Red O stain, the stained lipid droplets were dissolved in 100% isopropanol. The absorbance was measured at 490 nm.

### Morphological Assessment of Cell Apoptosis

Apoptosis was assessed by treating the cells with 10 µg/mL Hoechst 33342 for 30 min at 37°C, using the Hoechst staining kit from Beyotime Institute of Biotechnology, Shanghai, China, following the manufacturer’s instructions. Nuclear morphological alterations in the cells were subsequently examined using an Olympus fluorescence microscope (Tokyo, Japan) at an emission wavelength of 461 nm. Apoptotic cells were characterized by intense blue fluorescence, whereas normal cells exhibited weaker fluorescence.

### Glucose Uptake after Recombinant Lentivirus Transfection In Vitro

The protocol for 2-[^3^H] deoxyglucose (2-DOG) uptake has been previously documented (20). PVPACs underwent a 3-h serum-starvation period, following which they were stimulated by the addition of 2-[^3^H]DOG (0.1 Ci, final concentration of 0.1 mM) in Krebs-Ringer-phosphate HEPES (KRPH) buffer (comprising 10 mM HEPES, pH 7.4, 131.2 mM NaCl, 4.7 mM KCl, 1.2 mM MgSO_4_, 2.5 mM CaCl_2_, and 2.5 mM NaH_2_PO_4_) for a duration of 10 min at 37°C. The uptake was halted by washing the cells, specifically by rinsing the monolayer four times with 500–1,000 μL of ice-cold KRPH buffer. Subsequently, 300 μL of 0.05 M NaOH was added to each well and mixed using a plate mixer. The plate was incubated for 2 h at 37°C to facilitate cell lysis. Radioactivity associated with the cells was quantified using a liquid scintillation counter.

### Animal Model and Carotid Artery Contractility

Male C57BL/6J mice aged 10–12 wk were procured from Shandong University Experimental Animal Center (Jinan, China). All animals were maintained under specific pathogen-free conditions with consistent room temperature, humidity, and a 12-h light-dark cycle. The mice were provided ad libitum access to a standard chow diet and water until the surgical procedure.

To investigate the roles of SGLT-1 in glycolipid metabolism and vascular remodeling, the mice were randomly allocated to four groups: PBS (negative control, *n* = 10), Lv-SGLT1 (overexpression of SGLT1, *n* = 10), Lv-siSGLT1 (knockdown of SGLT1, *n* = 10), and Lv-GFP (GFP control, *n* = 10).

Anesthesia was induced by an intraperitoneal injection of 5% chloral hydrate (35 mg/100 g). The fur was epilated and disinfected with iodophor. A ventral midline incision measuring 4–5 mm was made in the neck. The left common carotid artery (LCCA) was exposed via blunt dissection. Subsequently, the LCCA was surrounded externally by a lentivirus-based thermosensitive in situ gel (TISG) composed of tri-distilled water, 22.5% Pluronic F-127, 2.5% Poloxamer 188, and lentiviral vector. After a brief interval, the incision was closed with surgical sutures. Finally, the mice were closely monitored until they sufficiently recovered in a chamber placed on a heating pad following surgery.

Similar to the study conducted by Zhang et al. (21), to analyze carotid artery vasoreactivity, the LCCA was cleaned of connective tissue and adipose, cut into segments, and mounted on a multichannel isometric myograph (Danish Myo Technology, Aarhus, Denmark). The arteries were equilibrated in Krebs solution (120.0 mmol/L NaCl, 5.0 mmol/L KCl, 25.0 mmol/L NaHCO_3_, 11.0 mmol/L glucose, 1.0 mmol/L KH_2_PO_4_, 1.2 mmol/L MgSO_4_, and 1.0 mmol/L MgCl_2_, pH 7.4) at 37°C for 20 min, and then contractile capacity was tested by exposure to 124.0 mmol/L KCl-containing buffer (15.7 mmol/L NaCl,124.0 mmol/L KCl, 1.8 mmol/L CaCl_2_, 1.0 mmol/L MgCl_2_, 5.6 mmol/L glucose, and 10.0 mmol/L HEPES, pH 7.4) for 3×. The contractile ability of the vessels was determined by exposure to norepinephrine (the final concentration fluctuated between 10^−9^ and 10^−5^ mol/L) and sodium nitroprusside (the final concentration fluctuated between 10^−7^ and 10^−3^ mol/L). Isometric tension was recorded by a PowerLab recording device (ADInstruments).

### Western Blotting

The cells were washed three times with PBS, followed by complete draining of residual PBS. Subsequently, they were treated with 200 μL of RIPA lysis buffer (Beyotime, Shanghai, China) containing 2 μL of phenylmethylsulfonyl fluoride (PMSF) and 4 μL of phosphatase inhibitor cocktail for 20 min. After incubation, the cells were gently scraped from the plate using a cold plastic cell scraper and carefully transferred to a precooled microfuge tube. The cells were then subjected to 15 s of sonication and subsequently centrifuged at 4°C for 20 min at 10,000 *g*. The resulting supernatant was aspirated and mixed with the loading buffer at a 5:1 ratio. The resulting mixture was heated to 95°C for 10 min. Finally, the protein and loading buffer mixture was stored at −80°C. Equivalent amounts of protein were separated by sodium dodecyl sulfate-polyacrylamide gel electrophoresis and subsequently transferred to PVDF membranes. The membranes were blocked with TBS-0.05% Tween-20 containing 5% skim milk for 1 h at room temperature. Following this, they were washed three times for 5 min each with TBS-0.05% Tween-20 and incubated overnight with primary antibodies at 4°C. After incubation, the membranes were washed and incubated with the corresponding horseradish peroxidase (HRP)-conjugated secondary antibody. Subsequently, the membranes were washed again and briefly exposed to an ECL detection reagent, followed by detection using a FluorChemQ System (ProteinSimple).

### Reverse Transcription Quantitative PCR

Cellular RNA was extracted from cultured PAPVCs following stimulation with TRIzol reagent (Invitrogen) in accordance with the manufacturer’s instructions. Subsequently, complementary DNA was synthesized using the Prime Script RT Master Mix Kit (Takara, Tokyo, Japan). Reverse transcription-quantitative PCR (RT-qPCR) was performed in duplicate using the SYBR Premix Ex TaqTM Kit (Takara, Japan). Primers for RT-qPCR analysis were designed based on previously published sequences (Table 1). RNA (1 μg) was then used for cDNA synthesis. The PCR thermocycler conditions were as follows: initial denaturation at 95°C for 10 min, followed by 40 cycles of denaturation at 95°C for 15 s, and annealing/extension at 60°C for 60 s. Data analysis was conducted using the 2^−ΔΔCt^ method, with normalization against β-actin expression.

**Table 1.** Real-time quantitative polymerase chain reaction primers used to quantify mRNA expression

Target	Forward	Reverse
*SGLT-1*	GTGCACCTGTACCGTTTGTG	TTGGAGTCCTCTGGGATGTC
*GLUT-1*	ATCATCGGTGTGTACTGCGG	AGCCAAACACCTGGGCAATA
*GLUT-4*	ATGGCTGTCGCTGGTTTCTC	AGGACCCATAGCATCCGCAA
*FABP4*	TCACCATCCGGTCAGAGAGTA	TCCTGTCGTCTGCGGTGATT
*VEGF-A*	GACAGACAGACAGACACCGC	CAGACCACGGCTACTACGGA

### Immunocytochemistry

Cultured PVPACs were seeded onto glass coverslips and allowed to grow to subconfluence. They were subsequently subjected to three 5-min rinses in PBS and fixed using 4% paraformaldehyde. Following fixation, cells were rinsed in PBS, permeabilized with 0.1% Triton X-100 for 3 min, and blocked in PBS containing 10% goat serum for 1 h at room temperature. Subsequently, the cells were incubated overnight with primary antibodies at 4°C in a dark humidified chamber. After rinsing with PBS, the cells were exposed to a rhodamine (TRITC)-conjugated secondary antibody (goat anti-mouse/rabbit antibody; diluted 1:100) for 15 min and counterstained with 1 μg/mL DAPI for 5 min. The slides were then subjected to three 5-min washes in PBST, mounted using a fluorescent mounting medium, and observed under a laser scanning confocal microscope.

### Histology and Immunohistochemistry

After dehydration and paraffin embedding, the tissues were sectioned at a thickness of 5 μm using a microtome and transferred onto glass slides. For histological analysis, tissue sections were deparaffinized and stained with hematoxylin and eosin (HE). To inhibit endogenous peroxidase activity, the sections were immersed in 3% H_2_O_2_ solution at room temperature for 30 min.

To expose the antigenic epitope, the sections were subjected to antigen retrieval using citric acid buffer (pH 6.0) in a microwave oven, cooled to room temperature, rinsed in PBS three times for 5 min each, and blocked in PBS containing 10% goat serum for 1 h at room temperature. Subsequently, the cells were incubated overnight with primary antibodies at 4°C in a humidified chamber. After rinsing with PBS, the slides were treated with the two-step plus Poly-HRP Anti Mouse/Rabbit IgG Detection System in a humidified chamber at room temperature for 30 min, followed by DAB staining. The slides were washed thrice with PBS for 2 min each. Finally, the tissues were counterstained with hematoxylin for 1–2 min, washed in PBS, and rinsed under running water for 30 min.

Dehydration was accomplished by sequential immersion in graded alcohol concentrations of 50%, 70%, 90%, and 100% and two changes of xylene. Finally, the slides were covered with Permount Mounting Medium, blocked with 5% bovine albumin, incubated with the primary antibody, and treated with the Polink-2 Plus Polymer HRP Detection System.

### Statistical Analysis

The results are presented as the mean ± standard deviation and were analyzed using GraphPad Prism software (version 5.0). It is important to note that all the experiments included in this study were conducted in triplicate, yielding consistent results. Statistical comparisons among multiple groups were performed using one-way analysis of variance (ANOVA), and ratio comparisons were assessed using chi-square analysis. Statistical significance was determined using a threshold of *P* values < 0.05.

## RESULTS

### Expression of SGLT-1 Protein after Recombinant Lentivirus Transfection In Vitro

**Figure 1.**
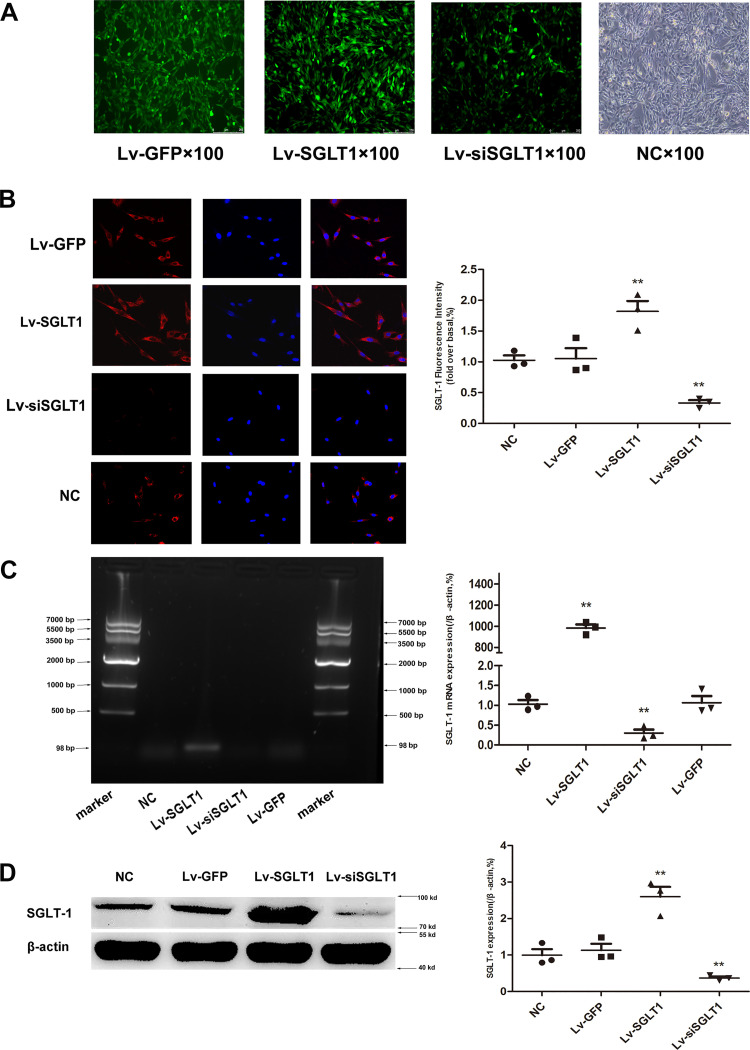
Identification of SGLT-1 expression after recombinant lentivirus transfection in vitro. *A*: seventy-two hours after recombinant lentivirus infection, morphology of PVPACs under bright-field (NC) and dark-field (Lv-GFP, Lv-SGLT1, and Lv-siSGLT1) (magnification, ×100). *B*: TRITC-labeled protein expression of SGLT-1 in immunocytochemistry staining (red) and DAPI (blue) staining was used to visualize nuclei (magnification, ×200). *C*: the mRNA expression of SGLT-1 in PVPACs after recombinant lentivirus transfection determined by AGE (*left* chart) and RT-qPCR (*right* chart), and normalized to that of β-actin (*right* chart). *D*: the expression of SGLT-1 in PVPACs after recombinant lentivirus transfection determined by Western blotting and normalized to that of β-actin. Data were expressed as means ± SD; the comparison of multiple groups was performed by ANOVA; **P < 0.01; vs. NC group. AGE, agarose gel electrophoresis; Lv-GFP, GFP control; Lv-SGLT1, SGLT-1 overexpression; Lv-siSGLT1, SGLT-1 knockdown; NC, normal control; PVPACs, perivascular preadipocytes; SGLT-1, sodium-glucose cotransporter 1; TRITC, rhodamine.

At 72 h after transfection with the recombinant lentivirus, nearly all PVPACs were successfully infected with the lentivirus, as depicted in Fig. 1*A*. SGLT-1 protein was primarily localized in the cytoplasm and cytomembrane, as illustrated in Fig. 1*B*.

Compared with the other experimental groups, the Lv-SGLT1 group (overexpression group) exhibited a notably elevated level of SGLT-1 mRNA expression, as demonstrated in Fig. 1*C*. Conversely, the Lv-siSGLT1 group (knockdown group) displayed reduced mRNA expression levels. This pattern was consistently observed in both immunofluorescence assays and Western blotting analyses. Specifically, after infection with recombinant lentivirus, SGLT-1 protein expression in the Lv-SGLT1 group was significantly higher than that in the other three groups, whereas SGLT-1 expression in the Lv-siSGLT1 group was lower than that in the other three groups, as depicted in Fig. 1, *B* and *D*.

### Effects of SGLT-1 on PVPACs Proliferation, Apoptosis, and Differentiation

**Figure 2.**
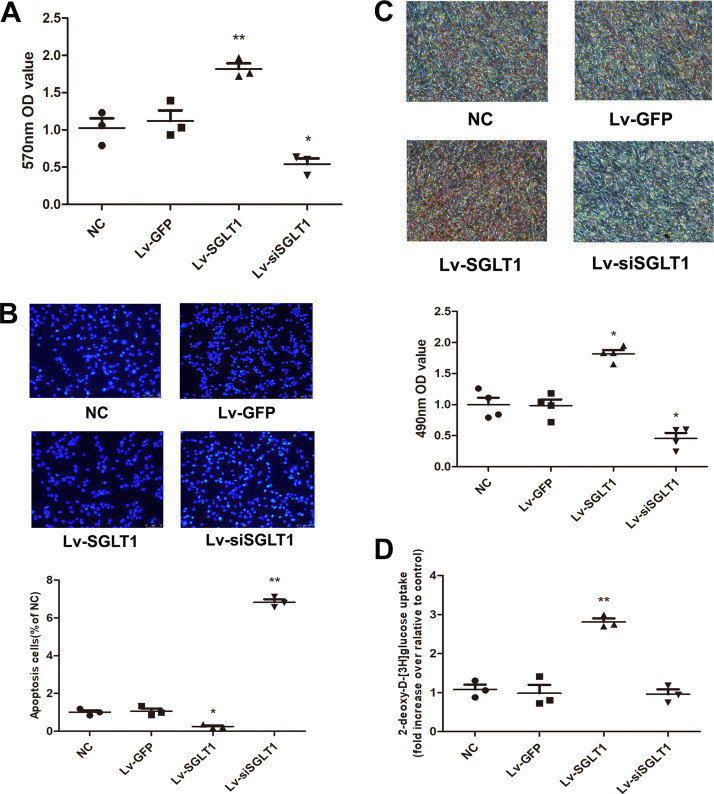
SGLT-1 promotes PVPACs’ proliferation, differentiation, glucose uptake, and inhibits its apoptosis. *A*: MTT assay indicated that SGLT-1 effectively stimulated PVPACs’ proliferation while SGLT-1 knockdown counteracted the effects. *B*: Hoechst 33342 staining revealed that SGLT-1 knockdown promoted PVPAC apoptosis while SGLT-1 overexpression counteracted the effects (magnification, ×100). *C*: Oil Red O staining on *day 10* after PVPACs’ differentiation (magnification, ×100). *D*: quantification of glucose uptake by 2-deoxy-d-[^3^H]glucose after recombinant lentivirus transfection in PVPACs. Data were expressed as means ± SD; the comparison of multiple groups was performed by ANOVA; *P < 0.05, **P < 0.01; vs. NC group. Lv-GFP, GFP control; Lv-SGLT1, SGLT-1 overexpression; Lv-siSGLT1, SGLT-1 knockdown; MTT, methyl thiazolyl tetrazolium; NC, normal control; PVPACs, perivascular preadipocytes; SGLT-1, sodium-glucose cotransporter 1.

PVPACs’ proliferation activity was calculated using the MTT assay (Fig. 2*A*). The Lv-SGLT1 group exhibited a significantly higher proliferative ratio and OD value than those of the other three groups. In contrast, the Lv-siSGLT1 group displayed a lower proliferation ratio and OD value than the other groups. These findings indicated that SGLT-1 effectively stimulated PVPACs’ proliferation. Conversely, Lv-siSGLT1 counteracted the proliferative effects of SGLT-1 on PVPACs.

To investigate the effect of SGLT-1 on the apoptosis of PVPACs, cellular staining with Hoechst 33342, a specialized fluorescent dye used to distinguish apoptotic cells from normal cells, was performed, and cellular morphology was observed using a fluorescence microscope. As shown in Fig. 2*B*, normal nuclei displayed diffuse homogeneous blue fluorescence, whereas apoptotic cells exhibited strong blue fluorescence. In the Lv-siSGLT1 group, typical morphological changes indicative of apoptosis were observed, including nuclear fragmentation, chromosomal condensation, and cell shrinkage (Fig. 2*B*). Conversely, the overexpression group (Lv-SGLT1 group) did not exhibit typical morphological changes associated with apoptosis, as depicted in Fig. 2*B*

On the 10th day after PVPACs differentiation, cellular differentiation capacity was evaluated via Oil Red O staining (Fig. 2*C*). The results demonstrated that overexpression of SGLT-1 protein promoted the adipogenic differentiation of PVPACs. As shown in Fig. 2*D*, compared with the control group, 2-deoxy-D-[^3^H]glucose uptake was notably increased in the Lv-SGLT1 group, whereas the glucose uptake level did not exhibit a noticeable difference in the Lv-siSGLT1 group.

### Expression of GLUT-1, GLUT-4, FABP4, and VEGF-A after Transfection in PVPACs

**Figure 3.**
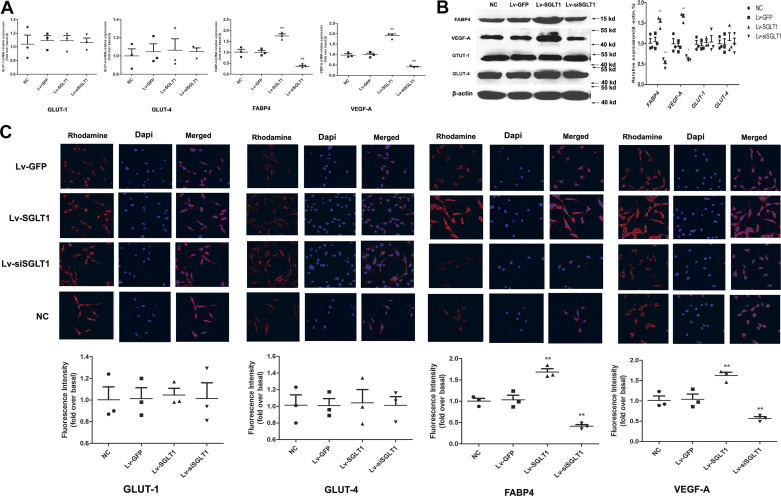
SGLT-1 promotes the expression of FABP4 and VEGF-A in PVPACs. *A*: RT-qPCR analysis revealed that SGLT-1 transfection increased the expression of FABP4 and VEGF-A, while there was no significant qualitative difference in the levels of membrane-associated GLUT-1 and GLUT-4. *B*: Western blot analysis showed that SGLT-1 overexpression promoted the expression of FABP4 and VEGF-A, and there was no significant qualitative difference in the levels of GLUT-1 and GLUT-4 between all groups. *C*: immunofluorescence analysis showed that SGLT-1 overexpression promoted the expression of FABP4 and VEGF-A (magnification, ×200). Data were expressed as means ± SD; the comparison of multiple groups was performed by ANOVA; **P < 0.01; vs. NC group. FABp4, fatty acid-binding protein 4; GLUT-1, glucose transporters 1; GLUT-4, glucose transporters 1; Lv-GFP, GFP control; Lv-SGLT1, SGLT-1 overexpression; Lv-siSGLT1, SGLT-1 knockdown; NC, normal control; PVPACs, perivascular preadipocytes; SGLT-1, sodium-glucose cotransporter 1; VEGF-A, vascular endothelial growth factor.

RT-qPCR analysis revealed that SGLT-1 promoted FABP4 and VEGF-A expression, whereas SGLT1 knockdown was associated with reduced FABP4 and VEGF-A expression (Fig. 3*A*). However, there were no significant qualitative differences in the expression levels of membrane-associated GLUT-1 and GLUT-4, as indicated in Fig. 3*A*.

Furthermore, Western blotting (Fig. 3*B*) and immunofluorescence analysis (Fig. 3*C*) confirmed that SGLT-1 overexpression and knockdown did not have a notable effect on the expression levels of GLUT-1 and GLUT-4 proteins. Conversely, SGLT-1 overexpression promoted the expression of FABP4 and VEGF-A, whereas SGLT1 knockdown exerted opposite effects, as observed in the results.

### SGLT-1 Activated Akt/mTOR/p70S6K Signaling Pathway

**Figure 4.**
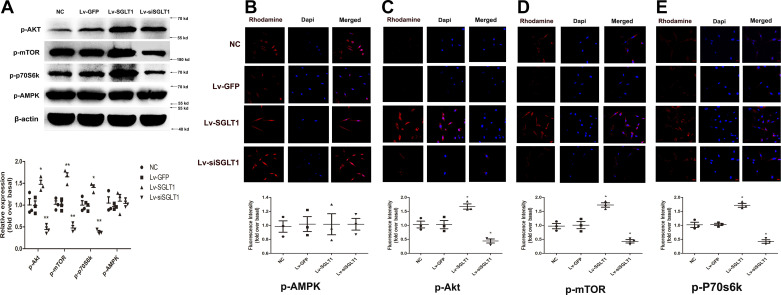
SGLT-1 activated Akt/mTOR/p70S6K signaling pathway in PVPACs. *A*: Western blot analysis revealed that SGLT-1 transfection activated Akt/mTOR/p70S6K signaling pathway. *B–E*: immunofluorescence analysis revealed that SGLT-1 gene transfection exhibited no differences in AMPK activity (phospho-Thr172 AMPK), but increased the phosphorylation levels of Akt/mTOR/p70S6K signaling pathway (magnification, ×200). Data were expressed as means ± SD; the comparison of multiple groups was performed by ANOVA; *P < 0.05, **P < 0.01; vs. NC group. AMPK, adenosine monophosphate-activated protein kinase; Lv-GFP, GFP control; Lv-SGLT1, SGLT-1 overexpression; Lv-siSGLT1, SGLT-1 knockdown; mTOR, mammalian target of rapamycin; NC, normal control; PVPACs, perivascular preadipocytes; SGLT-1, sodium-glucose cotransporter 1.

Western blot analysis indicated that SLC5A1 gene transfection did not result in any discernible differences in AMPK activity, as evidenced by the expression of phospho-Thr172 AMPK. However, it led to increased phosphorylation of Akt, mTOR, and p70S6K, as demonstrated in Fig. 4*A*. These results were consistent with the findings from immunofluorescence analysis, as shown in Fig. 4, *B*–*E*.

These results collectively suggest that SGLT-1 overexpression activates the Akt/mTOR/p70S6K signaling pathway but has no significant effect on the activation of AMPK. Consequently, it can be inferred that SGLT-1 executes its biological function in PVPACs primarily through the Akt/mTOR/p70S6K pathway, rather than the AMPK/mTOR pathway.

### Involvement of Akt/mTOR/p70S6K in SGLT-1-Induced PVPACs’ Proliferation and Differentiation

**Figure 5.**
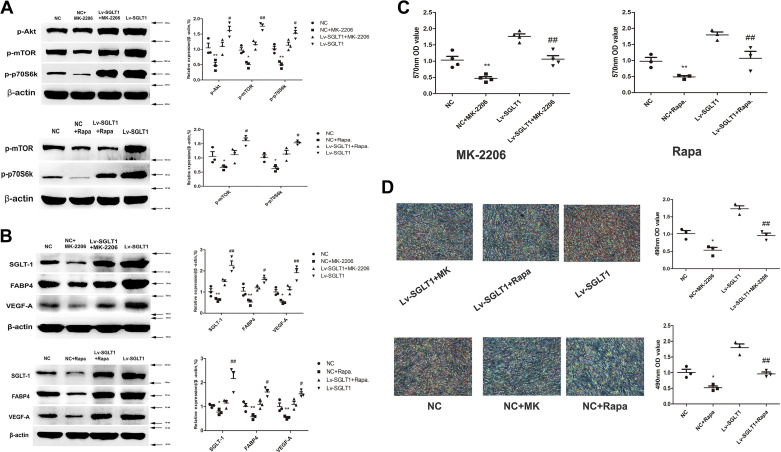
SGLT-1 stimulated PVPACs’ proliferation and differentiation by activating Akt/mTOR/p70S6K signaling pathway. *A*: Western blot analysis revealed that SGLT-1 overexpression activated Akt/mTOR/p70S6K signaling pathway could be blocked by signaling pathway inhibitors (MK-2206 and rapamycin). *B*: Western blot analysis revealed that MK-2206 and rapamycin, respectively, could inhibit the expression of FABP4 and VEGF-A and SGLT-1. *C*: MTT analysis revealed that MK-2206 and rapamycin, respectively, could attenuate PVPACs’ proliferation induced by SGLT-1. *D*: Oil-red O staining assays revealed that MK-2206 and rapamycin, respectively, could attenuate PVPACs’ differentiation induced by SGLT-1 versus inhibitor group (MK-2206 or rapamycin), #*P* < 0.05, ##*P* < 0.01; vs. inhibitor group (MK-2206 or rapamycin). *P < 0.05, **P < 0.01; vs. NC group. Lv-GFP, GFP control; Lv-SGLT1, SGLT-1 overexpression; Lv-siSGLT1, SGLT-1 knockdown; MTT, methyl thiazolyl tetrazolium; NC, normal control; PVPACs, perivascular preadipocytes; SGLT-1, sodium-glucose cotransporter 1.

To further corroborate the role of the Akt/mTOR/p70S6K signaling pathway in SGLT-1-induced PVPACs’ proliferation and adipogenesis, specific signaling pathway inhibitors were used in PVPACs treatment. Western blotting results demonstrated that SGLT-1-induced Akt phosphorylation was significantly inhibited by 5 μM MK-2206, a well-established inhibitor of Akt (Fig. 5*A*). Similarly, SGLT-1-induced mTOR and p70S6K activation was notably suppressed by 100 nM rapamycin, a commonly used inhibitor of mTOR (Fig. 5*A*).

Furthermore, Western blotting (Fig. 5*B*), MTT (Fig. 5*C*), and Oil Red O staining assays (Fig. 5*D*) collectively revealed that MK-2206 and rapamycin effectively counteracted the enhanced expression of FABP4 and VEGF-A induced by SGLT-1 and attenuated PVPACs’ proliferation and differentiation mediated by SGLT-1.

Based on these findings, it can be deduced that SGLT-1 induces PVPACs’ proliferation and differentiation by upregulating the Akt/mTOR/p70S6K signaling pathway. In addition, MK-2206 and rapamycin reversed SGLT-1-induced expression of FABP4 and VEGF-A, respectively (Fig. 5*B*). This suggests that SGLT-1 may influence glucolipid metabolism and angiogenesis via the Akt/mTOR/p70S6K signaling pathway. Furthermore, it is noteworthy that MK-2206 and rapamycin also downregulated SGLT-1 levels (Fig. 5*B*), implying a potential interaction between SGLT-1 and the Akt/mTOR/p70S6K signaling pathway.

### Expression of GLUT-1, GLUT-4, FABP4, and VEGF-A after Transfection In Vivo

**Figure 6.**
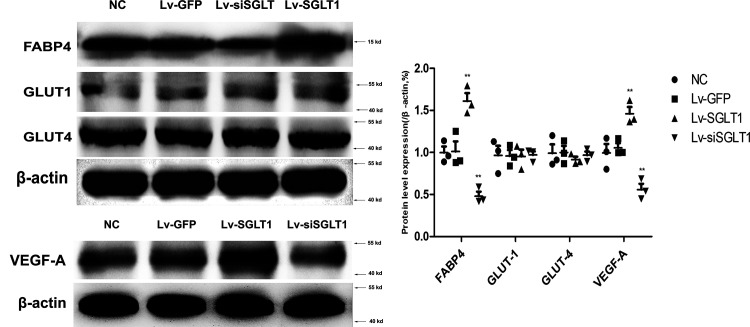
Expression of GLUT-1, GLUT-4, FABP4, and VEGF-A after transfection with lentivirus vector in vivo. Western blot analysis revealed that SGLT-1 overexpression promoted the expression of FABP4 and VEGF-A, but there was no difference in the levels of GLUT-1 and GLUT-4 between all groups in vivo. Data were expressed as means ± SD; the comparison of multiple groups was performed by ANOVA; **P < 0.01; vs. NC group. FABP4, fatty acid-binding protein 4; GLUT-1, glucose transporters 1; GLUT-4, glucose transporters 4; Lv-GFP, GFP control; Lv-SGLT1, SGLT-1 overexpression; Lv-siSGLT1, SGLT-1 knockdown; NC, normal control; SGLT-1, sodium-glucose cotransporter 1; VEGF-A, vascular endothelial growth factor.

**Figure 7.**
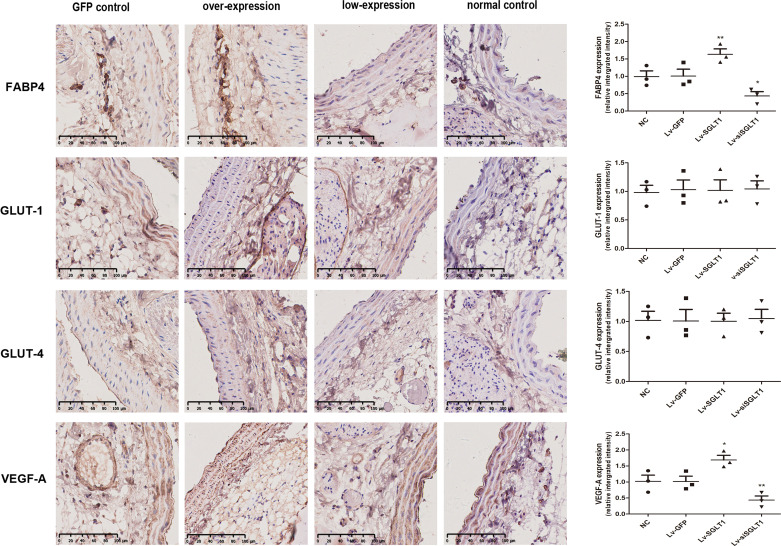
Immunohistochemical staining revealed that SGLT-1 overexpression promoted the expression of FABP4 and VEGF-A in vivo (magnification, ×100). Data were expressed as means ± SD; the comparison of multiple groups was performed by ANOVA; *P < 0.05, **P < 0.01; vs. NC group. Lv-GFP, GFP control; Lv-SGLT1, SGLT-1 overexpression; Lv-siSGLT1, SGLT-1 knockdown; NC, normal control; SGLT-1, sodium-glucose cotransporter 1.

Western blot analysis (Fig. 6, *A–C*) and immunohistochemical staining (Fig. 7) revealed that SGLT-1 overexpression led to an increase in the expression of FABP4 and VEGF-A not only in PVAT but also in the cells of the media and intima (Fig. 7). Conversely, SGLT-1 knockdown was associated with reduced FABP4 and VEGF-A expression. In summary, SGLT-1 overexpression increased the thickness of the entire vascular wall, leading to vascular remodeling. Conversely, SGLT-1 knockdown resulted in a reduction in vascular wall thickness and improvement in vascular remodeling (Fig. 7). Notably, there were no significant qualitative differences in the levels of membrane-associated GLUT-1 and GLUT-4, as previously mentioned.

### Effects of SGLT-1 on Adipogenesis and Vascular Remodeling In Vivo

**Figure 8.**
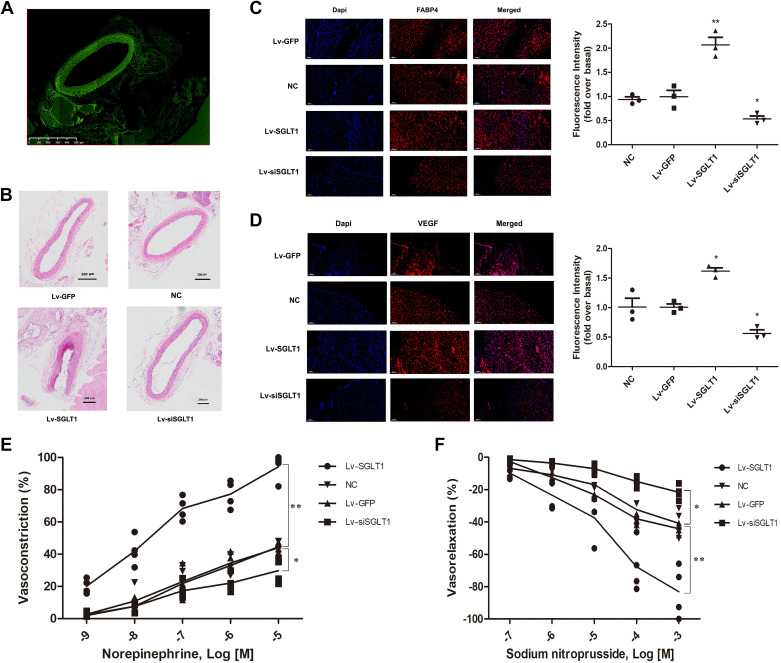
SGLT-1 promoted adipogenesis and vascular remodeling in vivo. *A*: GFP protein expression in the pericarotid area of mice is demonstrated by fluorescence microscope imaging system 2 wk after the injection of recombinant lentivirus (magnification, ×100). *B*: HE staining of the pericarotid area at 2 wk after the surgery indicated that SGLT-1 overexpression promoted vascular remodeling in vivo (magnification, ×100). *C* and *D*: immunofluorescence staining revealed that SGLT-1 overexpression promoted the expression of FABP4 and VEGF-A in vivo (magnification, ×100). *E* and *F*: vascular tension test verified that SGLT-1 overexpression enhances norepinephrine-induced carotid artery vasoconstriction and attenuates sodium nitroprusside-induced carotid artery vasodilation, SGLT-1 knockdown had the opposite effects. Data were expressed as means ± SD; the comparison of multiple groups was performed by ANOVA; *P < 0.05, **P < 0.01; vs. NC group. Lv-GFP, GFP control; Lv-SGLT1, SGLT-1 overexpression; HE, hematoxylin and eosin; Lv-siSGLT1, SGLT-1 knockdown; NC, normal control; SGLT-1, sodium-glucose cotransporter 1.

To further evaluate the effects of SGLT-1 on adipogenesis and vascular remodeling in vivo, lentivirus-based TISG was applied externally to the LCCA of the mice. Immunofluorescence staining was used to confirm the successful expression of GFP protein, validating the effective transfection of the pericarotid area with recombinant lentivirus, as expected (Fig. 8*A*). Representative histological sections stained for GFP are shown in Fig. 8*A*.

Hematoxylin and eosin staining was used to depict the morphological characteristics of the pericarotid area 2 wk after surgery (Fig. 8*B*). The adipogenic potential of PVPACs was assessed based on FABP4 expression intensity. Immunofluorescence staining demonstrated that the expression of FABP4, a marker of preadipocyte differentiation into mature adipocytes, was lowest in the Lv-siSGLT1 group and highest in the Lv-SGLT1 group (Fig. 8*C*).

The angiogenic capability of the PVPACs was evaluated based on the intensity of VEGF-A expression, a marker of angiogenesis. Immunofluorescence staining for VEGF-A revealed that the Lv-SGLT1 group exhibited a higher expression level than the Lv-GFP, Lv-siSGLT1, and NC groups (Fig. 8*D*). Conversely, the Lv-siSGLT1 group displayed the lowest fluorescence intensity compared with the Lv-SGLT1, Lv-GFP, and NC groups (Fig. 8*D*).

To evaluate the effects of SGLT-1 on vascular tension, we evaluated vasoconstriction caused by norepinephrine and vasodilation caused by sodium nitroprusside of carotid artery segments in all groups of mice. The study found that carotid artery vasoconstriction increased and vasodilation decreased in the Lv-SGLT1 group, while carotid artery contraction capacity decreased and relaxing ability increased in the Lv-siSGLT1 group (Fig. 8, *E* and *F*).

These findings indicate that SGLT-1 overexpression promotes carotid artery remodeling, whereas SGLT-1 knockdown ameliorates carotid artery remodeling.

## DISCUSSION

To our knowledge, there are no prior reports on SLC5A1 intervention in PVPACs’ biofunction. This study effectively confirms the biological role of SLC5A1 in regulating preadipocytes in the PVAT, along with its effective regulatory mechanism in vascular remodeling. The validation was conducted both in vivo and in vitro.

Unlike SGLT-2 (encoded by SLC5A2), SGLT-1 (encoded by SLC5A1) is widely distributed, with only a few exceptions (22). This study also verified the expression of SGLT-1 protein in PVPACs (Fig. 1). Notably, as a protein molecule involved in regulating glucose metabolism, SGLT-1 was shown to enhance glucose absorption (Fig. 2*D*) while also managing fat metabolism in PVPACs (Figs. 2*C*, 3, and 4, *C* and *D*), as anticipated. The classic role of SGLT-1 in the small intestine is the secondary and active transport of Na^+^-d-glucose through the apical membrane, exerting a rate-limiting influence on glucose absorption from dietary sources by the intestine (6). Individuals harboring SGLT-1 mutations exhibit diminished glucose transport capacity in the small intestine (6).

Furthermore, this study demonstrated the feasibility of modulating SLC5A1 gene expression through lentiviral vector-mediated overexpression and RNA interference technology-mediated low expression, both in vitro and in vivo (Figs. 2, 3, 4, 5, 6, 7, and 8). This targeted gene knockdown approach is more precise in intervening with SGLT than nonspecific SGLT inhibitors, such as the traditional drugs phlorizin and phloretin. To investigate the effect of SLC5A1 on the biological function of PVPACs and vascular remodeling in vivo, a novel TISG-carrying lentiviral vector was used in the left carotid artery region of mice. This innovative approach effectively limits lentivirus vector transfection to the local carotid artery area, mitigating its systemic effects (including intestinal mucosal cells) and reducing potential adverse effects on experimental results.

Consistent with the in vitro cell experiments (Figs. 1, 2, 3, 4, and 5), overexpression of the SLC5A1 gene promoted the expression of FABP4 and VEGF-A in PVPACs. This ultimately results in increased adipose tissue volume surrounding the carotid artery and thickening of various layers of the vascular wall, subsequently promoting carotid vascular remodeling (Figs. 6, 7, and 8). These findings underscore the influence of SLC5A1 gene expression on glucose uptake, adipogenic differentiation, and proliferative capacity of PVPACs. Moreover, they suggested a direct and/or indirect effect on the cellular function and structural remodeling of the vascular wall through glycolipid metabolism.

Several studies have confirmed that the functional regulation of SGLT-1 is a complex process involving both transcriptional and posttranscriptional mechanisms (23). SGLT-1 transport activity is influenced by various molecular regulators, including protein kinases (9). The regulation of SGLT-1 via protein kinase C may necessitate the activation of a complex signaling cascade that includes p38/MAPK, extracellular signal-regulated kinase (ERK)/MAPK, c-Jun N-terminal protein kinase (JNK)/MAPK, and phosphoinositide-3 kinase (PI3K)/Akt/mammalian target of rapamycin (mTOR) (24). Notably, the PI3K/Akt/mTOR signaling pathway is involved in governing cell survival, growth, proliferation, angiogenesis, apoptosis, and autophagy (11, 25). Dysregulation of this pathway has been observed in various human diseases, including diabetes, cardiovascular disease, cancer, and neurological disorders (26, 27). This study demonstrated that SGLT-1 overexpression enhances the phosphorylation of Akt and mTOR signaling proteins, which can be counteracted by the addition of the Akt-specific inhibitor MK-2206 or the mTOR-specific inhibitor rapamycin (Fig. 5, *A* and *B*). Simultaneously, MK-2206 or rapamycin reduced the expression of SGLT-1, Akt, and mTOR and diminished the expression of FABP4 (a marker of adipogenic differentiation) and VEGF-A (an angiogenic marker). This reduction in expression was accompanied by a decrease in the proliferation and adipose differentiation capacity of the PVPACs (Fig. 5, *C* and *D*). These findings suggest that the SGLT-1/Akt/mTOR/p70S6K signaling pathway may play a role in the regulation of PVPACs’ biofunctions.

Another group of signaling molecules involved in cellular energy metabolism include AMP-AMPK and glucose transporters. In eukaryotic cells, the primary glucose transporter proteins are GLUT-1 and GLUT-4. SGLT-1 has recently been identified as the primary glucose transporter protein overexpressed in the heart, along with GLUT-1 and GLUT-4 (28). AMPK, known as the “cell energy sensor,” primarily monitors cellular energy state (15). Sayour et al. propose a positive association between SGLT-1 and AMPK expression and left ventricular end-diastolic diameter, along with a negative correlation with left ventricular ejection fraction in mammalian species with cardiomyopathy (28, 29). However, Ramratnam et al. (30) conclusively demonstrated that SGLT-1 expression in mouse cardiomyocytes has no significant effect on the phosphorylation of AMPK molecules, contradicting the findings of Banerjee et al. (29), but aligned with the results of this study (Fig. 4). Surprisingly, contrary to our initial expectations, SGLT-1 protein had no significant effect on AMPK phosphorylation (Fig. 4), consistent with the findings of Ramratnam et al. (30). In addition, there is substantial controversy regarding whether SGLT-1 regulates GLUT-1 and GLUT-4 expression in the cardiovascular system (28, 29). Banerjee et al. discovered a significant increase in the mRNA expression of SGLT-1 in the cardiomyocytes of mice with glycogen-storage cardiomyopathy due to AMPK gene mutations. However, GLUT-1 and GLUT-4 expression was not significantly altered despite increased glucose uptake in cardiomyocytes (29). This result aligns with our findings (Figs. 3, 6, and 7). In summary, our research supports the notion that AMPK activity can influence SGLT-1 expression, but SGLT-1 has no significant effect on the activation of AMPK, GLUT-1, or GLUT-4 (Figs. 3, 4, 6, and 7).

A recent clinical study revealed that the dual SGLT-1/-2 inhibitor sotagliflozin reduces the overall risk of cardiovascular death, HF hospitalization, and emergency HF in patients with type 2 diabetes mellitus and chronic kidney disease (31). This suggests that SGLT-1 inhibition may offer additional benefits. Seidelmann et al. (32) reported that the heterozygosity of missense variants in SLC5A1, leading to reduced SGLT-1 function, was associated with a significant reduction in the risk of diabetes, HF, and cardiovascular death in patients. Li et al. (33) detected significant upregulation of SGLT-1 expression in a mouse model of myocardial infarction. Matsushita et al. (34) found that chronic pressure overload in mice (aortic coarctation model) results in cardiac hypertrophy and fibrosis by upregulating the expression of SGLT-1 and IL-18. Although the promoting effect of SGLT-1 on cell proliferation is universally acknowledged, whether it promotes or inhibits apoptosis remains disputed and may be cell type-dependent (35, 36). Our study indicates that SGLT-1 overexpression in PVPACs promotes proliferation and differentiation (Figs. 2, 3, 4, and 5) while facilitating local vascular remodeling (Figs. 6, 7, and 8). In contrast, SGLT-1 knockdown had the opposite effect on PVPACs and vascular remodeling, both in vitro and in vivo (Figs. 6, 7, and 8).

However, there are certain limitations to the current experimental setup. First, we applied a lentivirus vector carrying the SLC5A1 gene to the carotid artery region of mice for in vivo modeling. Although this approach avoids some of the drawbacks associated with methods such as tail vein or intraperitoneal injection in mice, it is worth noting that uncontrolled and indiscriminate lentivirus infection in the carotid artery region might still impact other tissues within the vascular wall, not limited to PVAT. This could potentially have affected the experimental results. Second, as previously mentioned, the regulation of SLC5A1 expression involves multiple mechanisms and signaling pathways, and this study focuses only on specific pathways. Third, the absence of SGLT-1 knockout may weaken the robustness of the results of the study. Fourth, a more comprehensive exploration of the interplay between glucose and lipid metabolism would strengthen the depth of the mechanistic exploration in this study. Fifth, the study lacked a comparison between the effects of SGLT-1 and SGLT-2 on the biological function and vascular remodeling of target cells and tissues. In addition, there have been no investigations into their potential synergistic effects or cooperative mechanisms. Although SGLT-1 significantly enhanced the expression of FABP4 and VEGF-A in PVPACs, suggesting a potential role in inducing glycolipid metabolism and angiogenesis, further studies are needed to confirm this hypothesis. Although subsequent research is required to investigate the underlying mechanisms, we underscore the significance of SGLT-1 in regulating adipogenesis and vascular remodeling in vivo, with the expectation that this will pave the way for new avenues of exploration into the etiology of atherosclerotic cardiovascular disease and vascular remodeling.

### Conclusions

In summary, we have proposed a novel perspective suggesting that SGLT-1 augments the proliferation, adipogenesis, and glucose uptake of PVPACs, suppresses cellular apoptosis, and promotes vascular remodeling through activation of the Akt/mTOR/p70S6K signaling pathways.

## DATA AVAILABILITY

The authors declare that any supporting data or material associated with this original research is available from corresponding author under reasonable request.

## GRANTS

This work was supported in part by grants from the Innovation Team Project of the “20 Regulations for Universities” funding Program of Jinan (No. 2021GXRC107), Science and Technology Innovation Project of Jinan City (No. 201602153; No. 202019193), Major Research and Development Project of Shandong Province (No. ZR2020MH041), Natural fund project of Shandong Province (No. GG201703080074), the project of the Central Government Guides Local Science and Technology Development (No. 2021Szvup073), and the National Natural Science Foundation of China (No. 81170274; No. 82170462).

## DISCLOSURES

No conflicts of interest, financial or otherwise, are declared by the authors.

## AUTHOR CONTRIBUTIONS

P.L. conceived and designed research; Z.L., J.W., and P.T. performed experiments; Z.L., J.W., P.T., Y.L., L.X., C.F., and X.H. analyzed data; Z.L. and P.L. interpreted results of experiments; Z.L. prepared figures; Z.L. and Y.L. drafted manuscript; Z.L. edited and revised manuscript; P.L. approved final version of manuscript.

## References

[1] Cui W, Niu Y, Sun Z, Liu R, Chen L. Structures of human SGLT in the occluded state reveal conformational changes during sugar transport. Nat Commun 14: 2920, 2023. doi:10.1038/s41467-023-38720-1. 37217492 PMC10203128

[2] Niu Y, Cui W, Liu R, Wang S, Ke H, Lei X, Chen L. Structural mechanism of SGLT1 inhibitors. Nat Commun 13: 6440, 2022. doi:10.1038/s41467-022-33421-7. 36307403 PMC9616851

[3] Han L, Qu Q, Aydin D, Panova O, Robertson MJ, Xu Y, Dror RO, Skiniotis G, Feng L. Structure and mechanism of the SGLT family of glucose transporters. Nature 601: 274–279, 2021. doi:10.1038/s41586-021-04211-w. 34880492 PMC9482448

[4] Cefalo CMA, Cinti F, Moffa S, Impronta F, Sorice GP, Mezza T, Pontecorvi A, Giaccari A. Sotagliflozin, the first dual SGLT inhibitor: current outlook and perspectives. Cardiovasc Diabetol 18: 20, 2019. doi:10.1186/s12933-019-0828-y. 30819210 PMC6393994

[5] Bode D, Semmler L, Wakula P, Hegemann N, Primessnig U, Beindorff N, Powell D, Dahmen R, Ruetten H, Oeing C, Alogna A, Messroghli D, Pieske BM, Heinzel FR, Hohendanner F. Dual SGLT-1 and SGLT-2 inhibition improves left atrial dysfunction in HFpEF. Cardiovasc Diabetol 20: 7, 2021. doi:10.1186/s12933-020-01208-z. 33413413 PMC7792219

[6] Mühlemann M, Zdzieblo D, Friedrich A, Berger C, Otto C, Walles H, Koepsell H, Metzger M. Altered pancreatic islet morphology and function in SGLT1 knockout mice on a glucose-deficient, fat-enriched diet. Mol Metab 13: 67–76, 2018. doi:10.1016/j.molmet.2018.05.011. 29859847 PMC6026318

[7] Neal B, Perkovic V, Mahaffey KW, de Zeeuw D, Fulcher G, Erondu N, Shaw W, Law G, Desai M, Matthews DR; CANVAS Program Collaborative Group. Canagliflozin and cardiovascular and renal events in type 2 diabetes. N Engl J Med 377: 644–657, 2017. doi:10.1056/NEJMoa1611925. 28605608

[8] Maccari R, Ottanà R. Sodium-glucose cotransporter inhibitors as antidiabetic drugs: current development and future perspectives. J Med Chem 65: 10848–10881, 2022. doi:10.1021/acs.jmedchem.2c00867. 35924548 PMC9937539

[9] Sano R, Shinozaki Y, Ohta T. Sodium–glucose cotransporters: functional properties and pharmaceutical potential. J Diabetes Investig 11: 770–782, 2020. doi:10.1111/jdi.13255. 32196987 PMC7378437

[10] Garcia-Ropero A, Santos-Gallego CG, Badimon JJ. SGLT receptors and myocardial ischaemia-reperfusion injury: inhibition of SGLT-1, SGLT-2, or both? Cardiovasc Res 115: 1572–1573, 2019. doi:10.1093/cvr/cvz077. 30873553

[11] Jiang Y, Liu P, Jiao W, Meng J, Feng J. Gax suppresses chemerin/CMKLR1‐induced preadipocyte biofunctions through the inhibition of Akt/mTOR and ERK signaling pathways. J Cell Physiol 233: 572–586, 2017. doi:10.1002/jcp.25918. 28326537

[12] Kim HW, Shi H, Winkler MA, Lee R, Weintraub NL. Perivascular adipose tissue and vascular perturbation/atherosclerosis. Arterioscler Thromb Vasc Biol 40: 2569–2576, 2020. doi:10.1161/ATVBAHA.120.312470. 32878476 PMC7577939

[13] Koenen M, Hill MA, Cohen P, Sowers JR. Obesity, adipose tissue and vascular dysfunction. Circ Res 128: 951–968, 2021. doi:10.1161/CIRCRESAHA.121.318093. 33793327 PMC8026272

[14] Angueira AR, Sakers AP, Holman CD, Cheng L, Arbocco MN, Shamsi F, Lynes MD, Shrestha R, Okada C, Batmanov K, Susztak K, Tseng Y-H, Liaw L, Seale P. Defining the lineage of thermogenic perivascular adipose tissue. Nat Metab 3: 469–484, 2021. doi:10.1038/s42255-021-00380-0. 33846639 PMC8136151

[15] Herzig S, Shaw RJ. AMPK: guardian of metabolism and mitochondrial homeostasis. Nat Rev Mol Cell Biol 19: 121–135, 2017. doi:10.1038/nrm.2017.95. 28974774 PMC5780224

[16] Yu JSL, Cui W. Proliferation, survival and metabolism: the role of PI3K/AKT/mTOR signalling in pluripotency and cell fate determination. Development 143: 3050–3060, 2016. doi:10.1242/dev.137075. 27578176

[17] Xu Z, Han X, Ou D, Liu T, Li Z, Jiang G, Liu J, Zhang J. Targeting PI3K/AKT/mTOR-mediated autophagy for tumor therapy. Appl Microbiol Biotechnol 104: 575–587, 2019. doi:10.1007/s00253-019-10257-8. 31832711

[18] Liu P, Feng J, Kong F, Lu Q, Xu H, Meng J, Jiang Y. Gax inhibits perivascular preadipocyte biofunction mediated by IGF-1 induced FAK/Pyk2 and ERK2 cooperative pathways. Cell Signal 26: 3036–3045, 2014. doi:10.1016/j.cellsig.2014.09.017. 25280940

[19] Chen J, Liu Y, Lu S, Yin L, Zong C, Cui S, Qin D, Yang Y, Guan Q, Li X, Wang X. The role and possible mechanism of lncRNA U90926 in modulating 3T3-L1 preadipocyte differentiation. Int J Obes (Lond) 41: 299–308, 2016. doi:10.1038/ijo.2016.189. 27780975 PMC5309343

[20] Yamamoto N, Ueda‐Wakagi M, Sato T, Kawasaki K, Sawada K, Kawabata K, Akagawa M, Ashida H. Measurement of glucose uptake in cultured cells. Curr Protoc Pharmacol 71: 12.14.1–12.14.26, 2015. doi:10.1002/0471141755.ph1214s71. 26646194

[21] Liu S, Jiang X, Lu H, Xing M, Qiao Y, Zhang C, Zhang W. HuR (Human Antigen R) regulates the contraction of vascular smooth muscle and maintains blood pressure. Arterioscler Thromb Vasc Biol 40: 943–957, 2020. doi:10.1161/ATVBAHA.119.313897. 32075416

[22] Vrhovac I, Balen Eror D, Klessen D, Burger C, Breljak D, Kraus O, Radović N, Jadrijević S, Aleksic I, Walles T, Sauvant C, Sabolić I, Koepsell H. Localizations of Na^+^-D-glucose cotransporters SGLT1 and SGLT2 in human kidney and of SGLT1 in human small intestine, liver, lung, and heart. Pflugers Arch 467: 1881–1898, 2014. doi:10.1007/s00424-014-1619-7. 25304002

[23] Koepsell H. The Na^+^-D-glucose cotransporters SGLT1 and SGLT2 are targets for the treatment of diabetes and cancer. Pharmacol Ther 170: 148–165, 2017. doi:10.1016/j.pharmthera.2016.10.017. 27773781

[24] Poulsen SB, Fenton RA, Rieg T. Sodium-glucose cotransport. Curr Opin Nephrol Hypertens 24: 463–469, 2015. doi:10.1097/MNH.0000000000000152. 26125647 PMC5364028

[25] Ersahin T, Tuncbag N, Cetin-Atalay R. The PI3K/AKT/mTOR interactive pathway. Mol Biosyst 11: 1946–1954, 2015. doi:10.1039/c5mb00101c. 25924008

[26] Yao H, Han X, Han X. The cardioprotection of the insulin-mediated PI3K/Akt/mTOR signaling pathway. Am J Cardiovasc Drugs 14: 433–442, 2014. doi:10.1007/s40256-014-0089-9. 25160498

[27] Yoshii A, Nagoshi T, Kashiwagi Y, Kimura H, Tanaka Y, Oi Y, Ito K, Yoshino T, Tanaka TD, Yoshimura M. Cardiac ischemia–reperfusion injury under insulin-resistant conditions: SGLT1 but not SGLT2 plays a compensatory protective role in diet-induced obesity. Cardiovasc Diabetol 18: 85, 2019. doi:10.1186/s12933-019-0889-y. 31262297 PMC6604374

[28] Sayour AA, Oláh A, Ruppert M, Barta BA, Horváth EM, Benke K, Pólos M, Hartyánszky I, Merkely B, Radovits T. Characterization of left ventricular myocardial sodium-glucose cotransporter 1 expression in patients with end-stage heart failure. Cardiovasc Diabetol 19: 159, 2020. doi:10.1186/s12933-020-01141-1. 32998746 PMC7528261

[29] Banerjee SK, Wang DW, Alzamora R, Huang XN, Pastor-Soler NM, Hallows KR, McGaffin KR, Ahmad F. SGLT1, a novel cardiac glucose transporter, mediates increased glucose uptake in PRKAG2 cardiomyopathy. J Mol Cell Cardiol 49: 683–692, 2010. doi:10.1016/j.yjmcc.2010.06.003. 20600102 PMC2932762

[30] Ramratnam M, Sharma RK, D'Auria S, Lee SJ, Wang D, Huang XYN, Ahmad F. Transgenic knockdown of cardiac sodium/glucose cotransporter 1 (SGLT1) attenuates PRKAG2 cardiomyopathy, whereas transgenic overexpression of cardiac SGLT1 causes pathologic hypertrophy and dysfunction in mice. J Am Heart Assoc 3: e000899, 2014. doi:10.1161/JAHA.114.000899. 25092788 PMC4310371

[31] Bhatt DL, Szarek M, Steg PG, Cannon CP, Leiter LA, McGuire DK, Lewis JB, Riddle MC, Voors AA, Metra M, Lund LH, Komajda M, Testani JM, Wilcox CS, Ponikowski P, Lopes RD, Verma S, Lapuerta P, Pitt B; SOLOIST-WHF Trial Investigators. Sotagliflozin in patients with diabetes and recent worsening heart failure. N Engl J Med 384: 117–128, 2021. doi:10.1056/NEJMoa2030183. 33200892

[32] Seidelmann SB, Feofanova E, Yu B, Franceschini N, Claggett B, Kuokkanen M, Puolijoki H, Ebeling T, Perola M, Salomaa V, Shah A, Coresh J, Selvin E, MacRae CA, Cheng S, Boerwinkle E, Solomon SD. Genetic variants in SGLT1, glucose tolerance, and cardiometabolic risk. J Am Coll Cardiol 72: 1763–1773, 2018. doi:10.1016/j.jacc.2018.07.061. 30286918 PMC6403489

[33] Li Z, Agrawal V, Ramratnam M, Sharma RK, D'Auria S, Sincoular A, Jakubiak M, Music ML, Kutschke WJ, Huang XN, Gifford L, Ahmad F. Cardiac sodium-dependent glucose cotransporter 1 is a novel mediator of ischaemia/reperfusion injury. Cardiovasc Res 115: 1646–1658, 2019. doi:10.1093/cvr/cvz037. 30715251 PMC6704393

[34] Matsushita N, Ishida N, Ibi M, Saito M, Sanbe A, Shimojo H, Suzuki S, Koepsell H, Takeishi Y, Morino Y, Taira E, Sawa Y, Hirose M. Chronic pressure overload induces cardiac hypertrophy and fibrosis via increases in SGLT1 and IL-18 gene expression in mice. Int Heart J 59: 1123–1133, 2018. doi:10.1536/ihj.17-565. 30101852

[35] Huang C-Y, Hsiao J-K, Lu Y-Z, Lee T-C, Yu LC-H. Anti-apoptotic PI3K/Akt signaling by sodium/glucose transporter 1 reduces epithelial barrier damage and bacterial translocation in intestinal ischemia. Lab Invest 91: 294–309, 2011. doi:10.1038/labinvest.2010.177. 20975661

[36] Lin N, Lin H, Yang Q, Lu W, Sun Z, Sun S, Meng L, Chi J, Guo H. SGLT1 inhibition attenuates apoptosis in diabetic cardiomyopathy via the JNK and p38 pathway. Front Pharmacol 11: 598353, 2020. doi:10.3389/fphar.2020.598353. 33597877 PMC7883645

